# Evaluation of a health systems knowledge translation network for Africa (KTNET): a study protocol

**DOI:** 10.1186/s13012-014-0170-4

**Published:** 2014-11-26

**Authors:** Elizabeth Ekirapa-Kiracho, David R Walugembe, Moses Tetui, Angela N Kisakye, Elizeus Rutebemberwa, Freddie Sengooba, Rornald M Kananura, Michel Wensing, Suzanne N Kiwanuka

**Affiliations:** School of Public Health, Makerere University, Kampala, Uganda; Scientific Institute for Quality in Healthcare, Radboud University Medical Centre, Nijmegen, The Netherlands

**Keywords:** Health systems, Knowledge translation, Evidence-based practice, Africa

## Abstract

**Background:**

Despite the increasing investment in health-related research in Sub-Saharan Africa, a large gulf remains between what is known and what is practiced in health systems. Knowledge translation programs aim to ensure that a wide range of stakeholders are aware of and use research evidence to inform their health and health-care decision-making. The purpose of this study is to provide insight into the impacts on capacity building for knowledge translation and knowledge translation activities by a coalition of eight research groups in Africa.

**Methods/design:**

We will use a mixed methods approach. Key informant interviews and document reviews will be employed to evaluate changes in knowledge translation capacity and to evaluate the effects of knowledge translation on potential users of research. Quarterly teleconferences will be done to evaluate the impacts of knowledge translation activities on users of research. Using website tracking, we will be able to explore the influence of knowledge translation networking and dynamics of the knowledge translation network.

**Discussion:**

We have adopted the dynamic knowledge transfer model and the Landry framework to come up with a framework for this study so as to explore the capacity of producers and users of research to generate, disseminate, and use research findings, while highlighting their strengths and weaknesses. This information will be useful for guiding implementers that seek to build capacity on knowledge translation so as to promote the utilization of research findings for informing programs, practice, and policy.

## Introduction

In Sub-Saharan Africa, there is an increasing investment in health research [1,2]. In addition, research from other regions can be relevant for decision-makers in this region. However, a large gap remains between what is known and what is practiced in health systems [3]. Indeed, stakeholders such as the World Health Organization have signaled that the adoption of research findings in developing countries is low and needs to be improved [4].

Knowledge translation (KT) programs aim to ensure that a wide range of stakeholders are aware of and use research evidence to inform their health and health-care decision-making [5]. KT aims to identify the best evidence as well as the pathways that make it easier for the targeted individuals and organizations to use research evidence in decisions and practices, ideally involving all relevant stakeholders, including patients, consumers, health-care providers, and policymakers. KT has been defined as the exchange, synthesis, and effective communication of reliable and relevant research results with a focus on promoting interaction among the producers and users of research, removing the barriers to research use, and tailoring information to different target audiences so that effective interventions are used more widely [6].

The need to understand how best to translate knowledge into practice focuses increasingly on dealing with information overload, because access to information is facilitated by modern information technology in many settings. In Africa, mobile devices with access to the Internet are widely used. Hence, careful selection, assessment, and prioritization of information are crucial for potential users of research. Moreover, building the capacity of producers of evidence to package and disseminate evidence to various audiences, as well as building the capacity of the users of this evidence to demand for, access, interpret, and apply evidence, is a crucial step in this process. According to Hamel and Schrecker [7], most researchers and research users in low- and middle-income countries have a low capacity for KT. This implies that a lot of research evidence remains unutilized in policymaking and practice. It is therefore important to develop and increase the KT capacity of researchers and decision-makers.

Previous studies have pointed to the impact of interaction and face-to-face contact between research producers and users for effective knowledge transfer [8]. It has been noted that the KT process has over time evolved from what was previously a one-way flow from producers of research to the users to a more interactive mode of linkage and exchange [9]. Studies revealed that such exchanges include formal and informal initiatives ranging from tailored products and messages, briefing events and forums, as well as other forms of partnership [10,11]. When applied in settings where people come together, drawn by similar interests and goals, such initiatives have a vast potential for facilitating knowledge uptake and behavior change [12]. However, little is known about how best to do this, particularly in low- and middle-income countries.

The study presented in this protocol aims to provide insight into the impacts on capacity building for KT and outcomes of KT activities of a coalition of eight research groups in Africa. The specific objectives are as follows: 1. to evaluate changes in KT capacity of partners in the research groups; 2. to explore the influence of networking and dynamics in the coalition on the KT achievements of the coalition partners; and 3. to evaluate the effects of KT activities on potential users of research, using the Landry (2001) framework.

### Description of the KT program and its background

Since 2002, The Netherlands Organization for Scientific Research (NWO) has supported researchers at universities and institutions internationally to conduct health-related research and to develop research capacity. In Africa, NWO has supported eight research groups to generate much needed research evidence around health systems in Africa. The research groups have focused on four areas: service delivery in obstetrics and child health, health-care financing (insurance initiatives), medical supplies and technologies (laboratory), and governance in health-care (accountability and community initiatives). The KT activities and their evaluation of the eight research groups have been organized in a separate project, called The Knowledge Translation Network Africa (KTNET Africa), which started in November 2013. This study protocol focuses on the evaluation of KT activities in this project. Table 1 provides details of the groups funded as well as their main research topics.

**Table 1:** **Research groups funded by NWO in Africa**

**Name of coalition**	**Country**	**Project title**
Malaria Elimination Project	Rwanda	Empowering the community towards malaria elimination
Accelerate Ghana	Ghana	Accelerating progress towards attainment of Millennium Development Goal 4 and 5 in Ghana through basic health system function strengthening
Community Based Health Insurance in Ethiopia	Ethiopia	Community Based Health Insurance in Ethiopia
Towards a client oriented health insurance system in Ghana	Ghana	Towards a client-oriented health insurance system in Ghana
Developing Sustainable Community Health Resources in Poor Settings in Uganda	Uganda	Developing Sustainable Community Health Resources in Poor Settings in Uganda
Improving maternal health services through political accountability mechanisms in Burundi and DR Congo	Burundi and DRC Congo	Improving maternal health services through political accountability mechanisms in Burundi and DR Congo
Maternal Health – South Africa/Rwanda	Rwanda and South Africa	Mainstreaming a health systems approach to delivery of emergency maternal health services: transdisciplinary research in Rwanda and South Africa
SOCIALAB	Senegal	Addressing Social, Cultural and Historical Factors Limiting the contribution of Medical Laboratory Services in West Africa

To promote the use of this research and ensure stakeholder engagement, a central office at Makerere University School of Public Health (MakSPH) will work with the different coalition partners to enable them to tailor their research outputs to the users’ interests, create opportunities for the users to adapt the products to their needs, and foster continued interactions between researchers and users so as to promote KT. Specifically, the secretariat will (1) build KT capacity among the eight partners, (2) support partner-specific KT activities across the network by providing technical support and supporting targeted stakeholder engagement small grants, and (3) host and coordinate platforms for KT across the network and with other partners globally.

#### Conceptual framework for the study

For purposes of this study, two overarching frameworks were selected to guide the evaluation: (1) the dynamic knowledge transfer capacity (DKTC) model [13] and (2) the Landry framework for research utilization [14]. The Landry framework (2001) will be used to evaluate the activities of the users of research for the different settings based on the country research contexts. The DKTC model will be used to assess and evaluate the capacities for KTNET coalition partners to achieve their KT goals. These two frameworks are briefly described below.

The DKTC model views the process of KT as happening within a system with certain enabling capacities. The model is used to identify the components required for social systems (researchers, governments, practitioners, communities) to generate, disseminate, and use new knowledge to meet their needs. Once these needs and knowledge exist, then the four core capacities will need to be present or developed. The capacities are (1) generative capacity, (2) disseminative capacity, (3) absorptive capacity, and (4) adaptive and responsive capacity. Generative capacity is the ability to produce, discover, or improve knowledge and the processes, technologies, products, and services. Disseminative capacity denotes the ability to contextualize, format, adapt, translate, and diffuse knowledge through a social and/or technological network and to build commitment from stakeholders. Absorptive capacity is defined as the ability to recognize the value of new external knowledge, assimilate it, and apply it to address relevant issues for a system’s stakeholders. Adaptive and responsive capacity refers to the ability to continuously learn and renew elements of the knowledge transferring system in use, for constant change and improvement.

The Landry framework is a theoretical model that explains the utilization of research as a process rather than a product in which a single study leads to decision-making. This has been adopted and will be used to categorize the effects of KT as follows: transmission, cognition, reference, effort, influence, and application to form the stages of a ladder for knowledge utilization. According to Landry (2001), the six stages of the framework are cumulative in that cognition builds on transmission, reference on cognition, effort on reference, influence on effort, and application on influence. This framework will be used to independently look at each stage of the ladder in order to describe the factors that lead researchers to research utilization [14].

**Figure 1:**
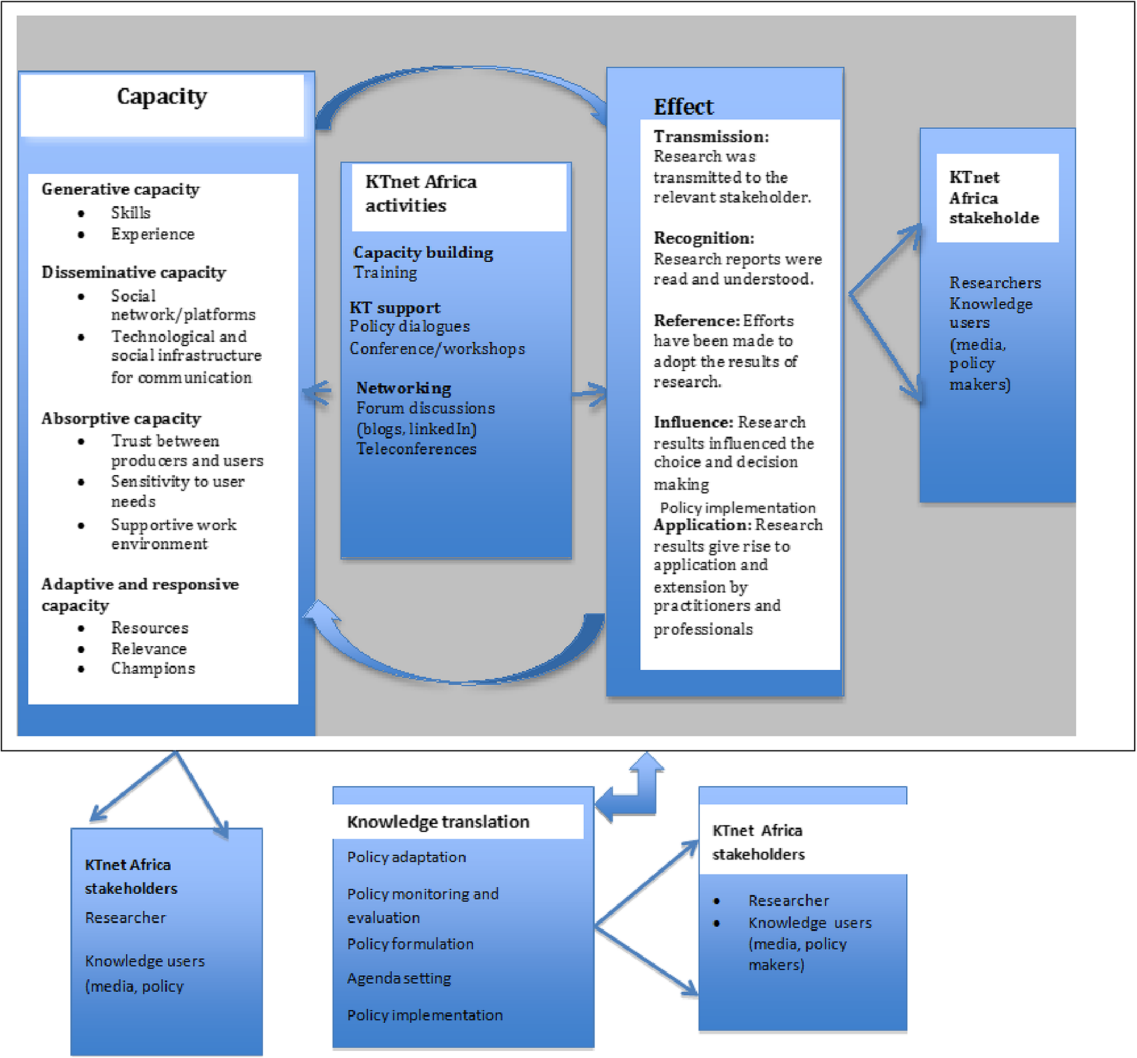
**The proposed conceptual framework for the KTNET Africa study and KT activities.**

Based on the KTNET objectives and the existing frameworks, an adaptation of the Landry and the DKTC models was used to guide the evaluation of the work. The proposed conceptual framework for the KTNET Africa study and KT activities (see Figure 1) consists of five dimensions that depict different features of the KT process. It highlights the capacity that researchers and users need to have in order to participate actively according to the DKTC model as well as the effects of KT on the users of the research based on the Landry framework. The KTNET Africa activities will contribute to the building of KT capacity while at the same time influence the use of research. The framework also illustrates that KT activities can take place during policy formulation, implementation, or evaluation process. Lastly, it also features the KTNET Africa stakeholders consisting of researchers and the users of the research.

## Methods

The program evaluation will be largely based on baseline and follow-up surveys and interviews in eight countries: Uganda, Rwanda, Senegal, Ghana, Ethiopia, Burundi, Democratic Republic of Congo, and South Africa. The study participants will be purposefully sampled and will include researchers (evidence producers), policymakers, media, civil society, health development partners, community members, health-care providers, and other potential evidence users. The questionnaires in the surveys will be composed of both structured items and semi-structured (open) questions. The number of participants that will be included in each of the eight countries will depend on the stakeholders relevant to the different coalitions depending on the results from the stakeholder analysis. In addition, we will collect information from written documents and online platforms. Table 2 describes the different data collection methods in relation to the three research objectives.

**Table 2:** **Data collection matrix**

**Objective**	**Method**	**Tools**	**Variables**
1. To evaluate changes in the knowledge translation capacity of the eight coalition partners	1. Key informant interview	1. Structured questionnaires	1. Number and quality of KT products produced
	2. Success story reports	2. Key informant guide	2. Participation in KT-related engagement activities
	3. Website tracking	3. Success story report guide	3. Contribution to decision-making by partners and policymakers
		Forum discussion networks	4. Citation of research results by other researchers
			5. Partners’ views in using research results
2. To explore the influence of KT networking and dynamics of the network on the KT achievements of the eight coalition partners	1. Website tracking and social network analysis	1. Free listing for Egos Internet-related contacts matrix	Density, centrality, connectedness
3. To evaluate the effects of KT activities on users of research using the Landry (2001) framework	1. Quarterly online/telephone survey	1. Questionnaire	Transmission
	2. Success stories	2. Success story report	Cognition
	3. KI interviews	3. KI interview guide	Reference
	4. Forum discussion	4. Forum discussion networks (LinkedIn, website)	Effort
			Influence
			Application

### Sampling procedures

#### Sampling procedures for key informant interviews

Purposive sampling techniques will be used to select respondents; we shall select coalition members and their partners who are likely to be influenced by KT interventions or who will influence KT interventions. For the qualitative interviews, initially, one key informant will be selected from each category, followed by more interviews until a point of saturation is reached. This selection will be guided by the relative importance of the stakeholder based on the perspective of the coalition partners.

#### Sampling procedures for the structured interviews

For the structured interviews, the snowballing sampling technique will be used to identify potential respondents (before the purposive sampling). The selected key informants will refer us to the partners they are interacting with. Additionally, we shall utilize the partners’ reports/records to identify other partners who benefited from KT interventions in different countries. The sampling will seek to include a diverse group of stakeholders. Table 3 shows the different categories of stakeholders who will be sampled. For the surveys, two, three, or four respondents will be selected from each category to ensure representativeness while keeping the numbers manageable. Approximately 15–20 respondents will be selected from each coalition depending on the stakeholders.

**Table 3:** **List of stakeholders**

**Stakeholder category**	**Estimated sample for structured questionnaire**	**Estimated sample for qualitative tool**
Researcher	3	1
Policymaker	2	1
Civil society organization	2	1
Health provider	3	1
Health insurance provider	2	1
Service user/client	4	1
Community representative	3	1
Media	2	1
NGO	2	1
Total for each coalition	15–20	9

### Data collection methods

Several data collection methods will be used to explore experiences and factors contributing to outcomes. The methods will include key informant interviews, review of documents and observations of KT activities forum discussion, social networking analysis, website tracking, and online surveys. Qualitative data will provide information that will contribute to answering objectives 1 to 3 that will assess progress in implementing the project, changes in KT capacity, KT dynamics, and KT effects.

#### Key informant interviews

To answer objectives 1 and 3, key informant interviews will be done at the beginning and at the end of the project. The key informant interviews will be used to measure changes in KT effects as well as participation in KT-related research. The number of key informants will depend on the coalition-specific project stakeholders as identified by the stakeholder analysis done at the beginning of the study. Key informants will include researchers, research users, media representatives, health service providers, and policymakers. All the key informants will be selected using purposive sampling techniques.

#### Document review

This will include review of success stories from coalition partners about the contribution of KT activities to the use of research results in decision-making by policymakers as well as other researchers and service delivery. They will also include review of forum discussions and blogs. Document review will be done throughout the project. This method will be used to answer part of objectives 1 and 3.

#### Website tracking

In order to explore the influence of KT networking and dynamics of the network among the eight coalition partners, Internet activity reports will be used and these will aim at indicating the number of people accessing KT Internet, documents/files downloaded, and documents/filed uploaded. This information will be captured through the monitoring system for the project, and it will be used to answer objective 2.

#### Quarterly teleconferences

Teleconferences will be done to evaluate the impacts of KT activities on users of research, using the Landry (2001) framework to structure the meetings. Partner KT activities will be tracked using minutes of the quarterly teleconferences. This will be done to answer objective 3. The detailed lists of indicators that will be collected are summarized in the monitoring and evaluation framework (Table 2).

### Data management

Pretesting will be done for all the instruments that will be used for data collection. All the interviews and key informant interviews will be done by well-trained and senior researchers after orientation about the study aims, objectives, and methods. KT website information access will be restricted. Website visitors will be required to have login user detail to access KT tools and participate in forum discussions as well as online surveys. This will help us to track how the documents on the website are used by people/visitors and partners. In addition, coalition contact persons and project staff involved in the project will meet monthly and quarterly to provide feedback and discuss the project progress. A team of advisors, as well as the research team, will provide regular oversight of the implementation of the study and provide advice.

### Data analysis

#### Objectives 1 and 3

The data to answer these objectives will be collected using key informant interviews. During the analysis, transcripts from key informant interviews will be transcribed and coded applying thematic content analysis, which identifies recurrent themes that form a cluster of linked categories containing similar meanings [15]. Coding of the data will be preceded by repeated reading of the data to facilitate familiarization of the data. The coding process will be guided by the research questions and conceptual framework. After the coding process is completed, the codes will be grouped into themes and subthemes, by sorting the different codes and collating them under potential themes. Arguments and explanations will be drawn and presented in relation to the research questions drawing upon the evidence, interpretation, and acknowledgement from researchers highlighting how the study may have influenced the research process and outcomes of the research for documents reviewed and minutes from quarterly teleconferences. The qualitative analysis will also be used to construct themes and explanations about network functioning, how influence is exercised in the network subgroup, or why cliques or other network structures get formed, expanded, or sustained. In addition, the qualitative analysis will explore the influence mechanisms that drive decision-makers’ perceptions, attitudes, and actions/decisions regarding research/influence information [16].

Document reviews will be done to assess how the KT capacity has changed as well as the effect of KT activities on research evidence use among the coalition partners. Information focusing on coalition partners who have disseminated their research results, stakeholders using research for policy planning or decision-making, and those who have knowledge of KT will be analyzed from the documents and presented using different methods. Data from document reviews will be used to provide more information for answering objectives 1 and 3 in addition to KIs.

#### Objective 2

In order to explore the influence of KT networking and dynamics of the network, website tracking of online discussion and website visitors will be done. Data will be analyzed using a social network analysis. This analysis will help us understand relationships and information flow between and among the coalition partners. We shall do this by studying interactions at three different levels—within the coalition itself, among the coalition partners and their stakeholders, and between the coalition partners from the different countries. This will be done through analysis of Internet-based interactions (websites, blogs, and discussion forums), interviews with coalition partners and their stakeholders, and lastly through review of relevant documents. To characterize the attributes of the networks, the information will be analyzed using both graphical and quantitative techniques. We will estimate several measures of network centrality as well as explore the density of the network [17]. The in-degree centrality for each individual stakeholder/partner will be estimated. In-degree centrality will help to measure the number of ties that are directed to a single partner/individual, and this will be calculated for each partner/individual in the network by adding together the number of times a partner or individual is mentioned by others. We will also explore the networks for existence of cliques (relatively isolated subnetworks) and boundary spanners (individuals who create links between cliques). We will use UCINET for the analysis of network data. UCINET has the capacity to graph network data for visualization and to perform block modeling [18].

## Discussion

The field of KT is expanding rapidly as evidence users are increasingly demanding researchers to increase the utilization of research evidence [14]. Previous literature on KT has focused on moving knowledge to use explaining the barriers to research utilization. Recent literature is more comprehensive, more sophisticated, and highly embedded in the actual contexts in which the knowledge application eventually occurs. The literature looks more at the institutional and social linkages critical to KT. Such linkages include adaptation of products by research users and dissemination efforts by researchers. Many models and frameworks have been proposed to provide guidance for KT planning [19]. A key feature of these models is that participation of both the researchers and users of research evidence is emphasized throughout the KT process. For instance, the Canadian Institutes of Health Research (CIHR) model of KT identifies opportunities within the research cycle at which the interactions, communities, and partnerships that can help facilitate KT could occur. It is augmented by three models and frameworks: (1) the interactive-focused framework, which offers a comprehensive approach to guide the interaction of knowledge creators and knowledge users; (2) the context-focused models and frameworks, which are very important in understanding the contextual factors that could play important roles in the success or failure of the KT efforts; and (3) the individual-focused models such as the Stetler Model of Research Utilization, developed by Stetler (2001), that was developed with the intention that it could be used by individual practitioners as a procedural and conceptual guide for the application of research in practice.

Although critical in planning for KT, the above models and frameworks have not explicitly taken into account the capacities required by the system to facilitate the use of research findings to influence policy, programs, and practice. Therefore, the DKTC model that focuses on the capacities that must be present in organizations and social systems as a precondition for knowledge transfer to occur has been proposed by Lavis et al. [8]. The Landry framework that was proposed by Landry et al. [14] and has been used to measure the utilization of knowledge produced by researchers was adopted for this study and combined with the DKTC model to form the conceptual framework.

The use of these two frameworks will allow us to explore the capacity of producers and users of research to generate, disseminate, and use research findings, while highlighting their strengths and weaknesses. Secondly, it will also allow a comprehensive analysis of the effects of various KT activities that will be implemented under the different research programs. This information will be useful for guiding implementers that seek to build capacity on KT so as to promote the utilization of research findings for informing programs, practice, and policy.

### Ethics

The study was approved by the Institutional Review Board of Makerere University School of Public Health and the Uganda National Council for Science and Technology. Written informed consent will be obtained for all the participants.

## Abbreviations

KT: Knowledge translation
NWO: The Netherlands Organization for Scientific Research
KTNET: Knowledge translation network
KTP: Knowledge translation platforms
MakSPH: Makerere University School of Public Health
MGD: Millennium development goal
DKTC: Dynamic knowledge transfer capacity model
MOU: Memorandum of understanding
